# Performance Analysis of UAV-Assisted Hybrid FSO/RF Communication Systems under Various Weather Conditions

**DOI:** 10.3390/s23177638

**Published:** 2023-09-03

**Authors:** Yan Wu, Dejin Kong, Qian Wang, Gang Li

**Affiliations:** The State Key Laboratory of New Textile Materials and Advanced Processing Technologies, School of Electronic and Electrical Engineering, Wuhan Textile University, Wuhan 430200, China; wuyan@wtu.edu.cn (Y.W.); djkou@wtu.edu.cn (D.K.); wqian@wtu.edu.cn (Q.W.)

**Keywords:** UAV-assisted hybrid FSO/RF communication systems, Exponentiated Weibull turbulence, Nakagami-m fading, pointing errors, receiver aperture, average bit error rate, outage probability

## Abstract

Nowadays, unmanned aerial vehicle (UAV) communication systems are commonly considered as one of the key enabling technologies for 6G. The hybrid free space optical (FSO)/radio frequency (RF) system has the advantages of both FSO and RF links to improve communication system performance, and the relay-assisted system adopts multi-hop transmission and cooperative diversity methods to extend communication coverage. Thus, a joint consideration of UAV-assistedUAV assisted relay in hybrid FSO/RF transmission is meaningful. In this paper, we aim to analyze the performance of UAV-assisted multi-hop parallel hybrid FSO/RF communication systems with and without pointing errors (PE) in terms of Bit Error Rate (BER) and outage probability. In our considered system, the FSO sub-link adopts the Exponential Weibull turbulence model and the RF sub-link suffers the Nakagami fading model. With these, new mathematical formulas of both BER and outage probability are derived under the UAV-assisted hybrid FSO/RF with different modulation methods. Through numerical evaluationnumerical simulations, the performances of UAV-assisted hybrid FSO/RF systems are analyzed under different weather conditions, modulation methods, optical receiver aperture, RF fading parameters, pointing errors, and relay structures. The results demonstrate that (1) compared to hybrid FSO/RF direct links, UAV-assisted hybrid FSO/RF systems can further improve system performance; (2) the performance of UAV-assisted hybrid FSO/RF systems varies with different relay structures; (3) large receiver aperture and RF fading parameters can further improve the communication performance of hybrid FSO/RF direct links and UAV-assisted hybrid FSO/RF systems.

## 1. Introduction

With the emergence of thousands of terminals, more communication resources are needed to demand users’ applications, such as interactive gaming, VR, etc. Traditional radio frequency (RF) communication methods will not satisfy those new demands anymore. Free space optical (FSO) communication systems are commonly used as one of telecommunications’ ‘last mile’ or next-generation mobile communication technology [1] because the characteristics of large capacity, fast network construction, license-free, and high security of free space optical (FSO) communication systems. However, the performance of ground FSO communication systems is greatly affected by atmospheric channels, such as the scattering effect of atmospheric particles (such as clouds, fog, rain, snow, aerosols, etc.) on the beam of light, causing attenuation of the light intensity on the receiver plane [2]. Atmospheric turbulence can cause scintillation, phase fluctuation, beam expansion, and beam wander on the receiver plane [3]. These all lead to limitations in the FSO communication, such as poor visibility and short transmission distance.

### 1.1. Related Works

In order to accurately analyze the impact of atmospheric turbulence on FSO communication systems, scholars have proposed many statistical decay models, including the Lognormal (LN) distribution [4], Negative Exponential distribution [5], κ distribution [6,7], Gamma–Gamma distribution [8,9], Málaga distribution [10], and Exponentiated Weibull (EW) distribution [11,12]. For weak turbulence, Parry et al. used LN distribution to model the probability density function of light irradiance. Negative Exponential and κ distributions are commonly used to simulate strong turbulent states. Andrews et al. proposed a modified Rytov theory, which led to the derivation of the Gamma–Gamma turbulence model. Due to its ability to simulate the changes from weak turbulence to strong turbulence, it has been widely used in the performance analysis of FSO systems. The Málaga distribution unifies most irradiance statistical models, including the LN distribution, κ distribution, and Gamma–Gamma distribution and is therefore considered a generalized turbulence model. Under all aperture averaging conditions, the EW distribution can provide good consistency with the probability density function (PDF) of light irradiance for simulation and experimental data in weak to strong turbulence and is commonly used to analyze the impact of aperture averaging technology on FSO system performance.

In order to mitigate the impact of atmospheric environment on the performance of ground FSO communication systems and improve the reliability and availability of wireless optical communication links, a mixed FSO/RF transmission system combining FSO links and millimeter wave radio frequency (MMW-RF) links has been proposed. Mixed FSO/RF communication systems are generally divided into mixed FSO/RF dual-hop systems and hybrid FSO/RF parallel transmission systems. The mixed FSO/RF dual-hop system is an asymmetric dual-hop relay system that can effectively expand coverage and reduce the impact of atmospheric environment on the FSO system [13]. Wang et al. [14] derived and analyzed the end-to-end average bit error rate (BER) expression for hybrid FSO/RF dual-hop systems based on the decode and forwarddecoding forwarding (DF) scheme under Exponential Weibull turbulence channels with nonzero boresight pointing error (PE) and Nakagami-m fading channels. The hybrid FSO/RF parallel transmission system is a cooperative communication system that can effectively improve communication system performance [15]. Odeyemi et al. [16] derived closed-form expressions of the bit error rate and outage probability of hybrid FSO/RF parallel systems based on a selective combination under Málaga turbulence with pointing errors and η−μ fading RF channels.

Due to the influence of turbulence, the performance of FSO communication systems deteriorates rapidly with the increase of transmission distance. A cooperative communication network based on multi-hop transmission (multi-hop relay-assisted network) is considered an effective solution to solve the problem [17]. Wang et al. [18] derived mathematical expressions of the end-to-end average bit error rate and outage probability of parallel multi-hop FSO systems under Exponential Weibull turbulence with nonzero boresight pointing errors. Zhang et al. [19] deduced the expression of the end-to-end average bit error rate and outage probability of the parallel multi-hop relay FSO system under Gamma–Gamma turbulence with a pointing error and different weather conditions and analyzed the impact of plane wave and spherical wave beams on the performance of parallel multi-hop relay FSO system.

In recent years, the development of unmanned aerial vehicle (UAV) technology has made UAV-assisted FSO communication a research hotspot for scientists [20,21,22,23]. UAV-assisted FSO communication is achieved by installing laser transmitters, receivers, and other devices on the UAV to communicate with ground terminals. The UAV has strong flexibility, convenient deployment, and wide coverage, which can effectively alleviate signal transmission problems in wireless optical communication and improve communication quality and reliability [24]. In practical applications, UAV-assisted FSO communication can be used in fields such as remote communication, monitoring data transmission, and disaster rescue. Lu et al. [25] analyzed and derived the outage probability of a UAV-based FSO dual-hop decode-and-forward system with multiple sources. Xu et al. [26] established and investigated a UAV-assisted mixed RF/FSO dual-hop communication system under the amplified-and-forward protocol with variable gain and derived the system metrics.

### 1.2. Motivation and Contributions

In order to simultaneously improve the communication performance, transmission distance, and coverage range of the communication system, this paper analyzes the performance of a UAV-assisted multi-hop parallel hybrid FSO/RF communication system (abbreviated as UAV-assisted hybrid system) that combines a hybrid FSO/RF parallel transmission system and a parallel multi-hop relay cooperative communication system by the UAV relay. Our main contributions in this work are pointed out as follows:

- To our knowledge, we, firstly, propose a multi-hop parallel hybrid FSO/RF communication system architecture with and without PE based on the UAV relay.
- New mathematical expressions for the end-to-end system in terms of average bit error rate and outage probability are derived under EW turbulence and Nakagami fading channels for four binary subcarrier modulation schemes.
- The effects of different weather environments, modulation methods, receiver apertures, RF fading parameters, pointing errors, and relay structures on the performance of our considered systems are analyzed through numerical evaluationnumerical simulations. As far as we know, no existing work considered the impact of weather environments and aperture averaging on UAV-assisted FSO communication.

The rest of the paper is structured as follows: in Section 2, the system model is presented. Section 3 analyzes the average bit error rate and outage probability. The performance of the hybrid FSO/RF direct link and UAV-assisted hybrid FSO/RF communication system is analyzed by system simulation in Section 4. Finally, conclusions are given in Section 5.

## 2. System and Channel Models

**Figure 1:**
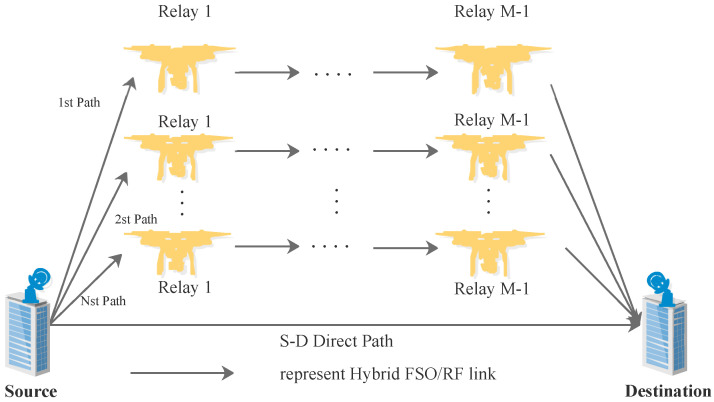
Structure of a UAV-assisted multi-hop parallel hybrid FSO/RF communication system.

We consider a UAV-assisted multi-hop parallel hybrid FSO/RF communication system, as shown in Figure 1, where there exist both a direct link and *N* numbers of hops between source and destination nodes, and each hop has M-1 relays for selections [27]. In any hop and direct link, the hybrid FSO/RF communication method based on DF relay protocol, subcarrier intensity modulation, and selective combination scheme are adopted. To avoid interferences, only one node is selected to relay and forward the signal to a next node at any hop. In order to improve the reliability of the communication system, the max–min path selection criterion is adopted [28].

### 2.1. One-Hop FSO SublinkSubsystem under Various Weather Conditions

Considering EW atmospheric turbulence only, according to Equation (8) in [29], the PDF of instantaneous signal to noise ratio (SNR) $\gamma_{x,y}^{FSO}$ without PE in a one-hop FSO sublinklink (the subscripts (x,y) denote the *y*-th hop hybrid FSO/RF link of the *x*-th path, especially (x,y) = (s,d) represent the direct hybrid FSO/RF link and (x,y) = (i,j) represent the *j*-th hop hybrid FSO/RF link of the *i*-th path) is:

(1) $$\begin{array}{cc} f_{\gamma_{x,y}^{FSO}}\left(\gamma_{x,y}^{FSO}\right) & =\frac{\alpha_{x,y}\beta_{x,y}}{2{\bar{\gamma }}_{x,y}^{FSO}\eta_{x,y}^{\beta_{x,y}}}{\left(\sqrt{\frac{\gamma_{x,y}^{FSO}}{{\bar{\gamma }}_{x,y}^{FSO}}}\right)}^{\beta_{x,y}-2}\exp\left[-{\left(\frac{1}{\eta_{x,y}}\sqrt{\frac{\gamma_{x,y}^{FSO}}{{\bar{\gamma }}_{x,y}^{FSO}}}\right)}^{\beta_{x,y}}\right]\\ & \times {\left\{1-\exp\left[-{\left(\frac{1}{\eta_{x,y}}\sqrt{\frac{\gamma_{x,y}^{FSO}}{{\bar{\gamma }}_{x,y}^{FSO}}}\right)}^{\beta_{x,y}}\right]\right\}}^{\alpha_{x,y}-1}, \end{array}$$

where the subscripts (x,y) denote a one-hop hybrid FSO/RF link, and (x,y) = (s,d) especially represent the direct hybrid FSO/RF link and (x,y) = (i,j) represent the *j*-th hop hybrid FSO/RF link of the *i*-th path. $\alpha_{x,y}$ and $\beta_{x,y}$ are the shape parameters of EW turbulence, and $\eta_{x,y}$ is the scale parameter. The values of these parameters are all greater than 0 and can be calculated according to [12]. ${\bar{\gamma }}_{x,y}^{FSO}={\left(P_{x,y}^{FSO}g_{x,y}^{FSO}R_{x,y}^{FSO}/\sigma_{x,y}^{FSO}\right)}^{2}$ is the average SNR of a one-hop FSO link [30], where $P_{x,y}^{FSO}$ is the transmittedtransmission power of the FSO link, $R_{x,y}^{FSO}$ is the sensitivity of the photodetector, $\sigma_{x,y}^{FSO}$ is the standard deviation of Gaussian white noise, and $g_{x,y}^{FSO}$ is the atmospheric loss of the FSO link.

Considering EW atmospheric turbulence and pointing errors [31,32], the PDF of instantaneous signal to noise ratio (SNR) $\gamma_{x,y}^{FSO}$ with PE in a one-hop FSO sublink is:

(2) $$\begin{array}{cc} f_{\gamma_{x,y}^{FSO}}\left(\gamma_{x,y}^{FSO}\right) & =\frac{\alpha_{x,y}\rho_{x,y}^{2}}{{\left(\eta_{x,y}A_{x,y}^{0}\right)}^{\rho_{x,y}^{2}}\gamma_{x,y}^{FSO}}{\left(\sqrt{\frac{\gamma_{x,y}^{FSO}}{{\bar{\gamma }}_{x,y}^{FSO}}}\right)}^{\rho_{x,y}^{2}}\sum_{t=0}^{\infty }\frac{{\left(-1\right)}^{t}\Gamma \left(\alpha_{x,y}\right)}{t!\Gamma \left(\alpha_{x,y}-t\right){\left(1+t\right)}^{1-\frac{\rho_{x,y}^{2}}{\beta_{x,y}}}}\\ & \times \;G_{1,2}^{2,0}\left(\frac{1+t}{{\left(\eta_{x,y}A_{x,y}^{0}\right)}^{\beta_{x,y}}}{\left(\sqrt{\frac{\gamma_{x,y}^{FSO}}{{\bar{\gamma }}_{x,y}^{FSO}}}\right)}^{\beta_{x,y}}\left|\begin{array}{c} 1\\ 0,1-\frac{\rho_{x,y}^{2}}{\beta_{x,y}} \end{array}\right.\right), \end{array}$$

where $\rho_{x,y}$ and $A_{x,y}^{0}$ are parameters related to pointing errors, $\rho_{x,y}=\omega_{x,y}^{eq}/2\sigma_{x,y}^{s}$ is the ratio of the equivalent beam radius to the standard deviation of the pointing error on the receiver plane, ${\left(\omega_{x,y}^{eq}\right)}^{2}={\left(\omega_{x,y}^{L}\right)}^{2}\sqrt{\pi }erfc\left(v_{x,y}\right)/\left[2v_{x,y}\exp\left(-v_{x,y}^{2}\right)\right]$, $\omega_{x,y}^{L}$ is the waist radius at the distance $L_{x,y}$ from the light source, $A_{x,y}^{0}={\left[erfc(v_{x,y})\right]}^{2}$ is the fraction of optical power received without pointing errors, $v_{x,y}=\left(\sqrt{\pi }d_{x,y}\right)/\left(\sqrt{2}\omega_{x,y}^{L}\right)$, $d_{x,y}$ is the radius of the receiver aperture, and erfc(·) is a complementary error function.

The atmospheric loss of the FSO link $g_{x,y}^{FSO}$ can be expressed by Beers Lamber Law [30], as follows:

(3) $$g_{x,y}^{FSO}=\left\{\begin{array}{c} \begin{array}{cc} \frac{A_{x,y}}{\pi {\left(\theta_{x,y}L_{x,y}/2\right)}^{2}}e^{-\left(\omega_{x,y}^{FSO}L_{x,y}\right)} & ,\;\mathrm{without}\;\mathrm{PE} \end{array}\\ \begin{array}{cc} e^{-\left(\omega_{x,y}^{FSO}L_{x,y}\right)} & ,\;\mathrm{with}\;\mathrm{PE} \end{array} \end{array}\right.,$$

where $\theta_{x,y}=2\omega_{x,y}^{L}/L_{x,y}$ is the beam divergence angle, $A_{x,y}=\left(\pi D_{x,y}^{2}\right)/4$, $D_{x,y}=2d_{x,y}$ is the receiver aperture diameter, $L_{x,y}$ is the beam propagation distance, and $\omega_{x,y}^{FSO}$ [dB/km] is the beam attenuation coefficient affected by the weather-dependent index of refraction structure parameter $C_{n}^{2}$ weather, as shown in Table 1 [30,33,34,35]. It is worth noting that when considering the pointing errors, the geometric loss of the beam during transmission has also been taken into account. By integrating Equation (1) with $F_{\gamma_{x,y}^{FSO}}\left(\gamma_{x,y}^{FSO}\right)=\int_{0}^{\gamma_{x,y}^{FSO}}f_{\gamma_{x,y}^{FSO}}\left(\gamma \right)d\gamma$$F_{\gamma_{}^{FSO}}\left(\gamma_{}^{FSO}\right)=\int_{0}^{\gamma_{}^{FSO}}f_{\gamma_{}^{FSO}}\left(x\right)dx$, the cumulative distribution function (CDF) of $\gamma_{x,y}^{FSO}$ without PE can be expressed as:

(4) $$F_{\gamma_{x,y}^{FSO}}\left(\gamma_{x,y}^{FSO}\right)={\left\{1-\exp\left[-{\left(\frac{1}{\eta_{x,y}}\sqrt{\frac{\gamma_{x,y}^{FSO}}{{\bar{\gamma }}_{x,y}^{FSO}}}\right)}^{\beta_{x,y}}\right]\right\}}^{\alpha_{x,y}}.$$

Similarly, according to Equation (2), the cumulative distribution function (CDF) of $\gamma_{x,y}^{FSO}$ with PE can be obtained as:

(5) $$F_{\gamma_{x,y}^{FSO}}\left(\gamma_{x,y}^{FSO}\right)=B_{x,y}\left(\gamma_{x,y}^{FSO}\right)\sum_{t=0}^{\infty }C_{x,y}G_{2,3}^{2,1}\left(D_{x,y}\left(\gamma_{x,y}^{FSO}\right)\left|\begin{array}{c} E_{x,y},1\\ 0,E_{x,y},E_{x,y} \end{array}\right.\right).$$

where $B_{x,y}\left(\gamma_{x,y}^{FSO}\right)=\frac{\alpha_{x,y}\rho_{x,y}^{2}}{\beta_{x,y}{\left(\eta_{x,y}A_{x,y}^{0}\right)}^{\rho_{x,y}^{2}}}{\left(\sqrt{\frac{\gamma_{x,y}^{FSO}}{{\bar{\gamma }}_{x,y}^{FSO}}}\right)}^{\rho_{x,y}^{2}}$, $C_{x,y}=\frac{{\left(-1\right)}^{t}\Gamma \left(\alpha_{x,y}\right)}{t!\Gamma \left(\alpha_{x,y}-t\right){\left(1+t\right)}^{1-\frac{\rho_{x,y}^{2}}{\beta_{x,y}}}}$, $D_{x,y}\left(\gamma_{x,y}^{FSO}\right)$$=\frac{1+t}{{\left(\eta_{x,y}A_{x,y}^{0}\right)}^{\beta_{x,y}}}{\left(\sqrt{\frac{\gamma_{x,y}^{FSO}}{{\bar{\gamma }}_{x,y}^{FSO}}}\right)}^{\beta_{x,y}}$, and $E_{x,y}=1-\frac{\rho_{x,y}^{2}}{\beta_{x,y}}$.

### 2.2. One-Hop RF SublinkSubsystem under Various Weather Conditions

In the one-hop RF sublink under the Nakagami-m fading channel, based on [33], the PDF of the SNR $\gamma_{x,y}^{RF}$ is:

(6) $$\begin{array}{cc} f_{\gamma_{x,y}^{RF}}\left(\gamma_{x,y}^{RF}\right) & ={\left(\frac{m_{x,y}}{{\bar{\gamma }}_{x,y}^{RF}}\right)}^{m_{x,y}}\frac{\gamma^{m_{x,y}-1}}{\Gamma (m_{x,y})}\exp\left(-\frac{m_{x,y}\gamma_{x,y}^{RF}}{{\bar{\gamma }}_{x,y}^{RF}}\right)\\ & ={\left(\frac{m_{x,y}}{{\bar{\gamma }}_{x,y}^{RF}}\right)}^{m_{x,y}}\frac{\gamma^{m_{x,y}-1}}{\Gamma (m_{x,y})}G_{0,1}^{1,0}\left[\frac{m_{x,y}\gamma_{x,y}^{RF}}{{\bar{\gamma }}_{x,y}^{RF}}\left|\begin{array}{c} -\\ 0 \end{array}\right.\right], \end{array}$$

where Γ(·) is a Gamma function, $G_{\cdot ,\cdot }^{\cdot ,\cdot }\left(\cdot \right)$ is a Meijer-G function, $m_{x,y}$ is the fading parameter ($m_{x,y}\geq 0.5$) of the one-hop RF link, and ${\bar{\gamma }}_{x,y}^{RF}=\frac{P_{x,y}^{RF}g_{x,y}^{RF}}{{\left(\sigma_{x,y}^{RF}\right)}^{2}}$ is the average SNR [36]. Herein, $P_{x,y}^{RF}$ is the transmittedtransmission power, and ${\left(\sigma_{x,y}^{RF}\right)}^{2}$ is the noise variance. At the frequency of 60 GHz (MMW-RF), the atmospheric loss of the RF link $g_{x,y}^{RF}$ can be expressed as [36]:

(7) $$g_{x,y}^{RF}\left[dB\right]=G_{x,y}^{t}+G_{x,y}^{r}-20\log_{10}\left(\frac{4\pi L_{x,y}}{\lambda_{x,y}^{RF}}\right)-L_{x,y}\left(\omega_{x,y}^{Oxg}+\omega_{x,y}^{Rain}\right),$$

where $G_{x,y}^{t}$ and $G_{x,y}^{r}$, respectively, represent the transmitter and receiver antenna gains of the RF channel, $\lambda_{x,y}^{RF}$ represents the RF carrier wavelength, and $\omega_{x,y}^{Oxg}$ and $\omega_{x,y}^{Rain}$ [dB/km], respectively, represent attenuation caused by oxygen absorption and rain, as shown in Table 1. The CDF of the SNR $\gamma_{x,y}^{RF}$ can be obtained through integration,

(8) $$F_{\gamma_{x,y}^{RF}}\left(\gamma_{x,y}^{RF}\right)=\frac{1}{\Gamma (m_{x,y})}G_{1,2}^{1,1}\left(\frac{m_{x,y}\gamma_{x,y}^{RF}}{{\bar{\gamma }}_{x,y}^{RF}}\left|\begin{array}{c} 1\\ m_{x,y},0 \end{array}\right.\right).$$

### 2.3. One-Hop Hybrid FS0/RF System Based on a Selective Combination Scheme

In a one-hop hybrid FSO/RF subsystem with a selective combination scheme, it detects the SNR of each sublink and selects the signal based on the maximum SNR. Therefore, the output SNR $\gamma_{x,y}^{SC}$ of the selection combiner on a one-hop link can be expressed as [37]:

(9) $$\gamma_{x,y}^{SC}=\max\left(\gamma_{x,y}^{FSO},\gamma_{x,y}^{RF}\right).$$

Therefore, the CDF of the SNR $\gamma_{x,y}^{SC}$ can be expressed as [37]:

(10) $$\begin{array}{cc} F_{\gamma_{x,y}^{SC}}\left(\gamma \right) & =\mathrm{Pr}(\max(\gamma_{x,y}^{FSO},\gamma_{x,y}^{RF})\leq \gamma )\\ & =\mathrm{Pr}(\gamma_{x,y}^{FSO}\leq \gamma ,\gamma_{x,y}^{RF}\leq \gamma )=F_{\gamma_{x,y}^{FSO}}\left(\gamma \right)F_{\gamma_{x,y}^{RF}}\left(\gamma \right). \end{array}$$

By substituting Equations (4) and (8) into Equation (10), the CDF of the output SNR $\gamma_{x,y}^{SC}$$\gamma_{i,j}^{SC}$ without PEat the j-th hop in i-th path can be obtained as:

(11) $$\begin{array}{c} F_{\gamma_{x,y}^{SC}}\left(\gamma \right)=\left\{\begin{array}{c} \begin{array}{cc} \frac{1}{\Gamma (m_{x,y})}G_{1,2}^{1,1}\left(\frac{m_{x,y}\gamma }{{\bar{\gamma }}_{x,y}^{RF}}\left|\begin{array}{c} 1\\ m_{x,y},0 \end{array}\right.\right){\left\{1-\exp\left[-{\left(\frac{1}{\eta_{x,y}}\sqrt{\frac{\gamma }{{\bar{\gamma }}_{x,y}^{FSO}}}\right)}^{\beta_{x,y}}\right]\right\}}^{\alpha_{x,y}} & ,\;\mathrm{without}\;\mathrm{PE} \end{array}\\ \begin{array}{cc} \frac{B_{x,y}\left(\gamma \right)}{\Gamma (m_{x,y})}\sum_{t=0}^{\infty }C_{x,y}G_{1,2}^{1,1}\left(\frac{m_{x,y}\gamma }{{\bar{\gamma }}_{x,y}^{RF}}\left|\begin{array}{c} 1\\ m_{x,y},0 \end{array}\right.\right)G_{2,3}^{2,1}\left(D_{x,y}\left(\gamma \right)\left|\begin{array}{c} E_{x,y},1\\ 0,E_{x,y},E_{x,y} \end{array}\right.\right) & ,\;\mathrm{with}\;\mathrm{PE} \end{array} \end{array}\right.. \end{array}$$

Similarly, the CDF of the output SNR $\gamma_{s,d}^{SC}$ of the combiner is rewritten as:

## 3. System Performance Analysis

According to the max–min criterion, the equivalent SNR $\gamma_{eq}^{\prime }$ of the optimal cooperative path from the source to the destination is denoted as [28]:

(12) $$\gamma_{eq}^{\prime }=\max_{i=1,\cdots ,N}\left(\min_{j=1,\cdots ,M}\left(\gamma_{i,j}\right)\right),$$

Therefore, the CDF of the equivalent SNR $\gamma_{eq}^{\prime }$ can be derived as:

(13) $$F_{{\gamma^{\prime }}_{eq}}\left(\gamma \right)={\left[1-\prod_{i=1}^{M}\left[1-F_{\gamma_{i,j}^{SC}}\left(\gamma \right)\right]\right]}^{N}.$$

For simplicity, consider that the channel of each hop follows an identical and independently distribution. Then, the above equation can be rewritten as

(14) $$\begin{array}{cc} F_{{\gamma^{\prime }}_{eq}}\left(\gamma \right) & ={\left[F_{\gamma_{eq_{i}}}\left(\gamma \right)\right]}^{N}={\left[1-{\left[1-F_{\gamma_{i,j}^{SC}}\left(\gamma \right)\right]}^{M}\right]}^{N}\\ & =\left\{\begin{array}{c} \begin{array}{cc} {\left\{1-{\left\{1-\frac{1}{\Gamma (m_{i,j})}G_{1,2}^{1,1}\left(\frac{m_{i,j}\gamma }{{\bar{\gamma }}_{i,j}^{RF}}\left|\begin{array}{c} 1\\ m_{i,j},0 \end{array}\right.\right){\left\{1-\exp\left[-{\left(\frac{1}{\eta_{i,j}}\sqrt{\frac{\gamma }{{\bar{\gamma }}_{i,j}^{FSO}}}\right)}^{\beta_{i,j}}\right]\right\}}^{\alpha_{i,j}}\right\}}^{M}\right\}}^{N} & ,\;\mathrm{without}\;\mathrm{PE} \end{array}\\ \begin{array}{cc} {\left[1-{\left[1-\frac{B_{i,j}\left(\gamma \right)}{\Gamma (m_{i,j})}\sum_{t=0}^{\infty }C_{i,j}G_{1,2}^{1,1}\left(\frac{m_{i,j}\gamma }{{\bar{\gamma }}_{i,j}^{RF}}\left|\begin{array}{c} 1\\ m_{i,j},0 \end{array}\right.\right)G_{2,3}^{2,1}\left(D_{i,j}\left(\gamma \right)\left|\begin{array}{c} E_{i,j},1\\ 0,E_{i,j},E_{i,j} \end{array}\right.\right)\right]}^{M}\right]}^{N} & ,\;\mathrm{with}\;\mathrm{PE} \end{array} \end{array}\right.. \end{array}$$

Furthermore, we can obtain the CDF of the system output SNR as [38]

(15) $$\begin{array}{cc} F_{\gamma }\left(\gamma \right) & =F_{\gamma_{s,d}^{SC}}\left(\gamma \right)\times F_{{\gamma^{\prime }}_{eq}}\left(\gamma \right)=F_{\gamma_{s,d}^{SC}}\left(\gamma \right)\times {\left[1-{\left[1-F_{\gamma_{i,j}^{SC}}\left(\gamma \right)\right]}^{M}\right]}^{N}\\ & =\left\{\begin{array}{c} \begin{array}{cc} \begin{array}{c} \frac{1}{\Gamma (m_{s,d})}G_{1,2}^{1,1}\left(\frac{m_{s,d}\gamma }{{\bar{\gamma }}_{s,d}^{RF}}\left|\begin{array}{c} 1\\ m_{s,d},0 \end{array}\right.\right){\left\{1-\exp\left[-{\left(\frac{1}{\eta_{s,d}}\sqrt{\frac{\gamma }{{\bar{\gamma }}_{s,d}^{FSO}}}\right)}^{\beta_{s,d}}\right]\right\}}^{\alpha_{s,d}}\times \\ {\left\{1-{\left\{1-\frac{1}{\Gamma (m_{i,j})}G_{1,2}^{1,1}\left(\frac{m_{i,j}\gamma }{{\bar{\gamma }}_{i,j}^{RF}}\left|\begin{array}{c} 1\\ m_{i,j},0 \end{array}\right.\right){\left\{1-\exp\left[-{\left(\frac{1}{\eta_{i,j}}\sqrt{\frac{\gamma }{{\bar{\gamma }}_{i,j}^{FSO}}}\right)}^{\beta_{i,j}}\right]\right\}}^{\alpha_{i,j}}\right\}}^{M}\right\}}^{N} \end{array} & ,\;\mathrm{without}\;\mathrm{PE} \end{array}\\ \begin{array}{cc} \begin{array}{c} \frac{B_{s,d}\left(\gamma \right)}{\Gamma (m_{s,d})}\sum_{t=0}^{\infty }C_{s,d}G_{1,2}^{1,1}\left(\frac{m_{s,d}\gamma }{{\bar{\gamma }}_{s,d}^{RF}}\left|\begin{array}{c} 1\\ m_{s,d},0 \end{array}\right.\right)G_{2,3}^{2,1}\left(D_{s,d}\left(\gamma \right)\left|\begin{array}{c} E_{s,d},1\\ 0,E_{s,d},E_{s,d} \end{array}\right.\right)\times \\ {\left[1-{\left[1-\frac{B_{i,j}\left(\gamma \right)}{\Gamma (m_{i,j})}\sum_{t=0}^{\infty }C_{i,j}G_{1,2}^{1,1}\left(\frac{m_{i,j}\gamma }{{\bar{\gamma }}_{i,j}^{RF}}\left|\begin{array}{c} 1\\ m_{i,j},0 \end{array}\right.\right)G_{2,3}^{2,1}\left(D_{i,j}\left(\gamma \right)\left|\begin{array}{c} E_{i,j},1\\ 0,E_{i,j},E_{i,j} \end{array}\right.\right)\right]}^{M}\right]}^{N} \end{array} & ,\;\mathrm{with}\;\mathrm{PE} \end{array} \end{array}\right.. \end{array}$$

### 3.1. Average Bit Error Rate

For UAV-assisted hybrid systems, the binary modulation scheme is used in FSO or RF transmission. Therefore, according to [16,39], the average BER can be mathematically expressed as:

(16) $$P_{b}=\frac{q^{p}}{2\Gamma (p)}\int_{0}^{\infty }{\left(\gamma \right)}^{p-1}\exp\left(-q\gamma \right)F_{\gamma }\left(\gamma \right)d\gamma ,$$

where *p* and *q* are parameters used to describe different binary modulation schemes, as shown in Table 2.

By substituting Equation (15) into Equation (16), the average BER of our proposed hybrid scheme without PE can be obtained:

(17) $$\begin{array}{cc} P_{b}^{RA} & =\frac{q^{p}}{2\Gamma (p)}\int_{0}^{\infty }\;{\left(\gamma \right)}^{p-1}\exp\left(-q\gamma \right)\frac{1}{\Gamma (m_{s,d})}G_{1,2}^{1,1}\left(\frac{m_{s,d}\gamma }{{\bar{\gamma }}_{s,d}^{RF}}\left|\begin{array}{c} 1\\ m_{s,d},\;0 \end{array}\right.\right){\left\{1-\exp\left[-{\left(\frac{1}{\eta_{s,d}}\sqrt{\frac{\gamma }{{\bar{\gamma }}_{s,d}^{FSO}}}\right)}^{\beta_{s,d}}\right]\right\}}^{\alpha_{s,d}}\\ & \;\times {\left\{1-{\left\{1-\frac{1}{\Gamma (m_{i,j})}G_{1,2}^{1,1}\left(\frac{m_{i,j}\gamma }{{\bar{\gamma }}_{i,j}^{RF}}\left|\begin{array}{c} 1\\ m_{i,j},0 \end{array}\right.\right){\left\{1-\exp\left[-{\left(\frac{1}{\eta_{i,j}}\sqrt{\frac{\gamma }{{\bar{\gamma }}_{i,j}^{FSO}}}\right)}^{\beta_{i,j}}\right]\right\}}^{\alpha_{i,j}}\right\}}^{M}\right\}}^{N}d\gamma . \end{array}$$

According to Equation (3.6.1) in [40], we have the approximate solution for the Generalized Gaussian–Laguerre quadrature function: $\int_{a}^{\infty }{\left(x-a\right)}^{c}\exp\left(-b(x-a)\right)f\left(x\right)dx\approx \sum_{\tau =1}^{n}w_{\tau }f\left(x_{\tau }\right)$, where $w_{\tau }$ and $x_{\tau }$ are the weight and a special point called the abscissa, respectively. Note that $w_{\tau }$ and $x_{\tau }$ are both determined by parameters a,b,c, and *n*. When a=b=1, it can be seen from [41] that $x_{\tau }$ is the τ-th root of the Generalized Laguerre polynomial $L_{n}^{\left(-1/2\right)}\left(x\right)$, the corresponding weight coeffificient $w_{\tau }=\Gamma \left[n+(1/2)\right]x_{t}/\left\{n!{\left(n+1\right)}^{2}{\left[L_{n+1}^{\left(-1/2\right)}\left(x_{t}\right)\right]}^{2}\right\}$. Therefore, Equation (17) can be simplified as:

(18) $$\begin{array}{cc} P_{b}^{RA} & =\frac{q^{p}}{2\Gamma (p)}\sum_{\tau =1}^{n}w_{\tau }\frac{1}{\Gamma (m_{s,d})}G_{1,2}^{1,1}\left(\frac{m_{s,d}x_{\tau }}{{\bar{\gamma }}_{s,d}^{RF}}\left|\begin{array}{c} 1\\ m_{s,d},0 \end{array}\right.\right){\left\{1-\exp\left[-{\left(\frac{1}{\eta_{s,d}}\sqrt{\frac{x_{\tau }}{{\bar{\gamma }}_{s,d}^{FSO}}}\right)}^{\beta_{s,d}}\right]\right\}}^{\alpha_{s,d}}\\ & \;\times {\left\{1-{\left\{1-\frac{1}{\Gamma (m_{i,j})}G_{1,2}^{1,1}\left(\frac{m_{i,j}\gamma }{{\bar{\gamma }}_{i,j}^{RF}}\left|\begin{array}{c} 1\\ m_{i,j},0 \end{array}\right.\right){\left\{1-\exp\left[-{\left(\frac{1}{\eta_{i,j}}\sqrt{\frac{\gamma }{{\bar{\gamma }}_{i,j}^{FSO}}}\right)}^{\beta_{i,j}}\right]\right\}}^{\alpha_{i,j}}\right\}}^{M}\right\}}^{N}d\gamma . \end{array}$$

Similarly, the average BER of our proposed hybrid scheme with PE can be derived as:

(19) $$\begin{array}{c} P_{b}^{RA}=\frac{q^{p}}{2\Gamma (p)}\sum_{\tau =1}^{n}w_{\tau }\frac{B_{s,d}\left(x_{\tau }\right)}{\Gamma (m_{s,d})}\sum_{t=0}^{\infty }C_{s,d}G_{1,2}^{1,1}\left(\frac{m_{s,d}x_{\tau }}{{\bar{\gamma }}_{s,d}^{RF}}\left|\begin{array}{c} 1\\ m_{s,d},0 \end{array}\right.\right)G_{2,3}^{2,1}\left(D_{s,d}\left(x_{\tau }\right)\left|\begin{array}{c} E_{s,d},1\\ 0,E_{s,d},E_{s,d} \end{array}\right.\right)\times \\ {\left[1-{\left[1-\frac{B_{i,j}\left(x_{\tau }\right)}{\Gamma (m_{i,j})}\sum_{t=0}^{\infty }C_{i,j}G_{1,2}^{1,1}\left(\frac{m_{i,j}x_{\tau }}{{\bar{\gamma }}_{i,j}^{RF}}\left|\begin{array}{c} 1\\ m_{i,j},0 \end{array}\right.\right)G_{2,3}^{2,1}\left(D_{i,j}\left(x_{\tau }\right)\left|\begin{array}{c} E_{i,j},1\\ 0,E_{i,j},E_{i,j} \end{array}\right.\right)\right]}^{M}\right]}^{N}. \end{array}$$

When M=N=0, Equations (18) and (19) are reduced to the BER for the hybrid FSO/RF direct link without PE and with PE, respectively.

### 3.2. Outage Probability

Outage probability is an important metric to evaluate the probability whether the receiver can successfully decode the message. Commonly, it is mathematically defined as the probability that the end-to-end output SNR is under a specific threshold $\gamma_{th}$. Therefore, we have [38]

(20) $$P_{out}=\mathrm{Pr}\left(\gamma <\gamma_{th}\right)=\int_{0}^{\gamma_{th}}f_{\gamma }\left(\gamma \right)d\gamma =F_{\gamma }\left(\gamma_{th}\right).$$

By considering Equation (15), we have the outage probability $P_{out}^{RA}$ of the system, i.e., $P_{out}^{RA}$ as

(21) $$\begin{array}{c} P_{out}^{RA}=\left\{\begin{array}{c} \begin{array}{cc} \begin{array}{c} \frac{1}{\Gamma (m_{s,d})}G_{1,2}^{1,1}\left(\frac{m_{s,d}\gamma_{th}}{{\bar{\gamma }}_{s,d}^{RF}}\left|\begin{array}{c} 1\\ m_{s,d},0 \end{array}\right.\right){\left\{1-\exp\left[-{\left(\frac{1}{\eta_{s,d}}\sqrt{\frac{\gamma_{th}}{{\bar{\gamma }}_{s,d}^{FSO}}}\right)}^{\beta_{s,d}}\right]\right\}}^{\alpha_{s,d}}\times \\ {\left\{1-{\left\{1-\frac{1}{\Gamma (m_{i,j})}G_{1,2}^{1,1}\left(\frac{m_{i,j}\gamma_{th}}{{\bar{\gamma }}_{i,j}^{RF}}\left|\begin{array}{c} 1\\ m_{i,j},0 \end{array}\right.\right){\left\{1-\exp\left[-{\left(\frac{1}{\eta_{i,j}}\sqrt{\frac{\gamma_{th}}{{\bar{\gamma }}_{i,j}^{FSO}}}\right)}^{\beta_{i,j}}\right]\right\}}^{\alpha_{i,j}}\right\}}^{M}\right\}}^{N} \end{array} & ,\;\mathrm{without}\;\mathrm{PE}, \end{array}\\ \begin{array}{cc} \begin{array}{c} \frac{B_{s,d}\left(\gamma_{th}\right)}{\Gamma (m_{s,d})}\sum_{t=0}^{\infty }C_{s,d}G_{1,2}^{1,1}\left(\frac{m_{s,d}\gamma_{th}}{{\bar{\gamma }}_{s,d}^{RF}}\left|\begin{array}{c} 1\\ m_{s,d},0 \end{array}\right.\right)G_{2,3}^{2,1}\left(D_{s,d}\left(\gamma_{th}\right)\left|\begin{array}{c} E_{s,d},1\\ 0,E_{s,d},E_{s,d} \end{array}\right.\right)\times \\ {\left[1-{\left[1-\frac{B_{i,j}\left(\gamma_{th}\right)}{\Gamma (m_{i,j})}\sum_{t=0}^{\infty }C_{i,j}G_{1,2}^{1,1}\left(\frac{m_{i,j}\gamma_{th}}{{\bar{\gamma }}_{i,j}^{RF}}\left|\begin{array}{c} 1\\ m_{i,j},0 \end{array}\right.\right)G_{2,3}^{2,1}\left(D_{i,j}\left(\gamma_{th}\right)\left|\begin{array}{c} E_{i,j},1\\ 0,E_{i,j},E_{i,j} \end{array}\right.\right)\right]}^{M}\right]}^{N} \end{array} & ,\;\mathrm{with}\;\mathrm{PE}. \end{array} \end{array}\right. \end{array}$$

By combing Equations (11) and (20), the outage probability of the hybrid direct link can be

(22) $$\begin{array}{c} P_{out}^{SD}=\left\{\begin{array}{c} \begin{array}{cc} \frac{1}{\Gamma (m_{s,d})}G_{1,2}^{1,1}\left(\frac{m_{s,d}\gamma_{th}}{{\bar{\gamma }}_{s,d}^{RF}}\left|\begin{array}{c} 1\\ m_{s,d},0 \end{array}\right.\right){\left\{1-\exp\left[-{\left(\frac{1}{\eta_{s,d}}\sqrt{\frac{\gamma_{th}}{{\bar{\gamma }}_{s,d}^{FSO}}}\right)}^{\beta_{s,d}}\right]\right\}}^{\alpha_{s,d}} & ,\;\mathrm{without}\;\mathrm{PE}, \end{array}\\ \begin{array}{cc} \frac{B_{s,d}\left(\gamma_{th}\right)}{\Gamma (m_{s,d})}\sum_{t=0}^{\infty }C_{s,d}G_{1,2}^{1,1}\left(\frac{m_{s,d}\gamma_{th}}{{\bar{\gamma }}_{s,d}^{RF}}\left|\begin{array}{c} 1\\ m_{s,d},0 \end{array}\right.\right)G_{2,3}^{2,1}\left(D_{s,d}\left(\gamma_{th}\right)\left|\begin{array}{c} E_{s,d},1\\ 0,E_{s,d},E_{s,d} \end{array}\right.\right) & ,\;\mathrm{with}\;\mathrm{PE}. \end{array} \end{array}\right. \end{array}$$

## 4. Numerical Results

The performance of our proposed system and the hybrid direct system are evaluated under different conditions, i.e., weather, receiver apertures, modulation methods, RF fading parameters, pointing errors, and network structures. The parameters of the UAV-assisted hybrid system and atmospheric channel are shown in Table 3, some of which are also adopted in [30,33,34,35]. When τ and *t* are both selected as 30*t* = 30, the approximate solution of the generalized Gaussian–Laguerre quadrature function tends to converge by simulations, and the average BER of the UAV-assisted hybrid system can be obtained according to Equations (18) and (19). Similarly, when *t* is chosen to be 30, the outage probability of a UAV-assisted hybrid system can be obtained according to Equation (21). For simplification, let the transmission power of FSO and RF links be the same, and each link has the same distance of 1km1km in simulations. The structure parameters (N=1,M=3), (N=5,M=3), (N=3,M=5), (N=3,M=2) have been selected to avoid entanglement.

**Figure 2:**
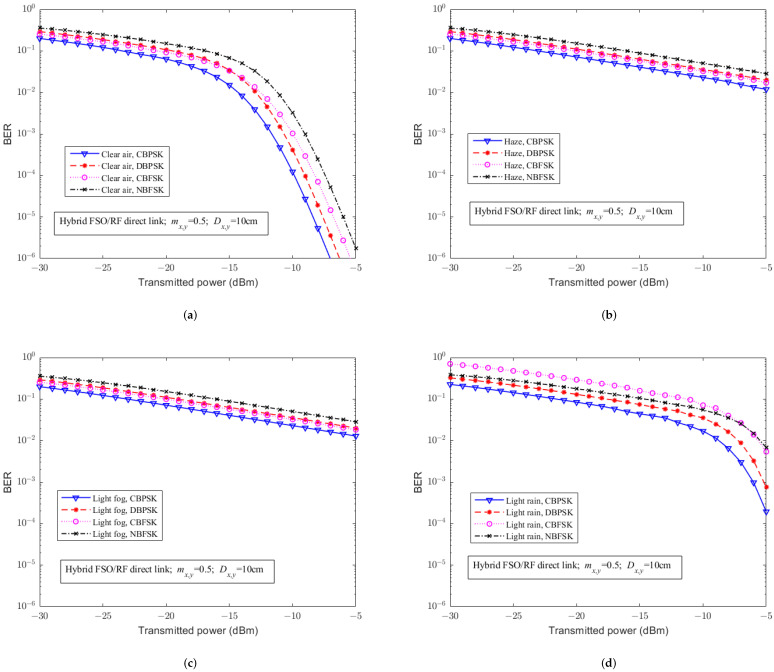
The relationship between BER performance and transmitted powertransmission of the hybrid FSO/RF direct link without PE under different weather conditions and modulation schemes. (**a**) Clear air. (**b**) Haze. (**c**) Light fog. (**d**) Light rain.

**Figure 3:**
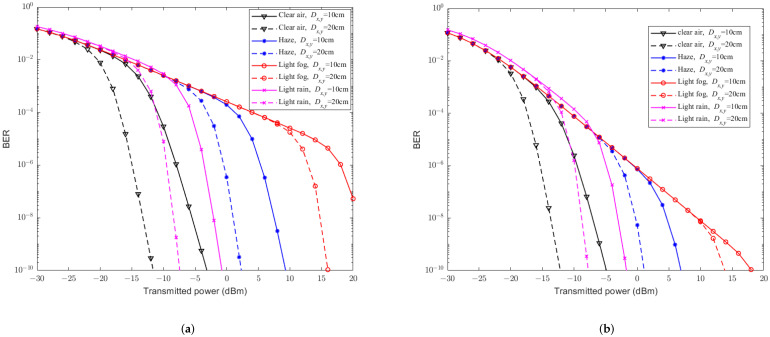
The relationship between the BER performance and transmittedtransmission power of hybrid FSO/RF direct links without PE. (**a**) $m_{x,y}=1$m=1. (**b**) $m_{x,y}=2$*m* = 2.

**Figure 4:**
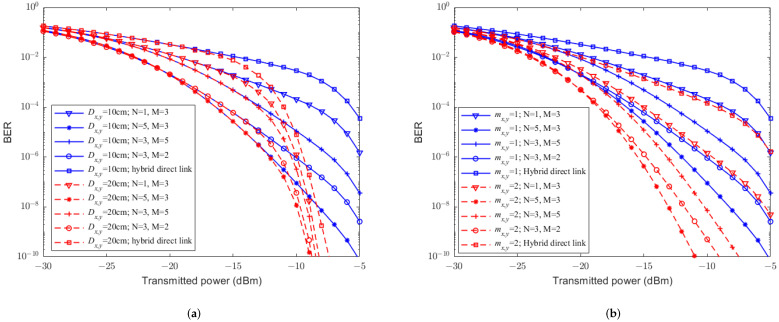
The relationship between the BER performance and transmittedtransmission power of UAV-assisted hybrid systems and the hybrid direct link without PE. (**a**) $m_{x,y}=1$m=1. (**b**) $D_{x,y}=10$ cm.

**Figure 5:**
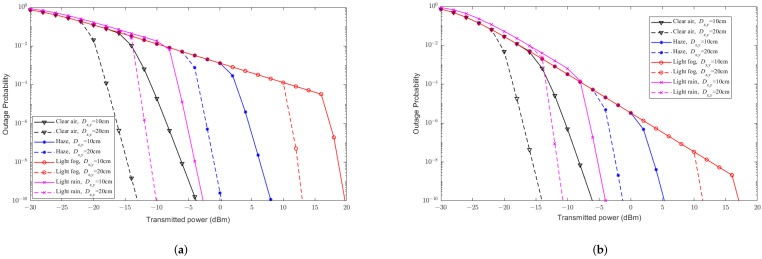
The relationship between outage probability and transmittedtransmission power of hybrid FSO/RF direct links without PE. (**a**) $m_{x,y}=1$m=1. (**b**) $m_{x,y}=2$m=2.

**Figure 6:**
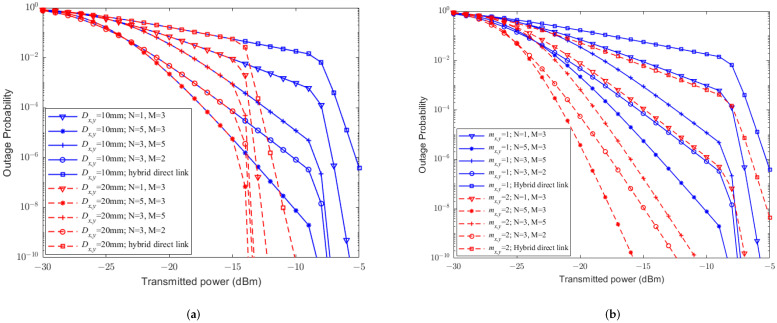
The relationship between outage probability and transmittedtransmission power of UAV-assisted hybrid systems and hybrid FSO/RF direct links without PE. (**a**) $m_{x,y}=1$m=1. (**b**) $D_{x,y}=10$ cm.

For simplicity, Figure 2, Figure 3, Figure 4, Figure 5 and Figure 6 mainly discuss the impact of weather on the performance of the system, without considering pointing errors. Figure 2 illustrates the effects of different weather conditions (i.e., clear air, haze, light fog, and light rain) on the BER of the hybrid FSO/RF direct link under different modulation schemes. From Figure 2, it can be seen that compared to the weather conditions of clear air and light rain weather, haze and light fog weather have a more severe impact on the BER of the hybrid direct link. The trend of the BER of hybrid direct links under haze and light fog weather conditions behave similarity, where for each single condition, CBPSK is the best, while NBFSK is the worst one in terms of their BER. Besides, based on Figure 2a, when the transmittedtransmission power is less than −15 dBm−15 dBm, the BER of the CBFSK is superior than that of the DBPSK scheme, while the performance of the CBFSK is gradually behind the DBPSK scheme when the transmittedtransmission power is larger than −15 dBm. Based on Figure 2d, when the transmittedtransmission power is less than −7 dBm−7 dBm, the BER of the NBFSK is superior than that of the CBFSK scheme, while the performance of the NBFSK is gradually behind the CBFSK scheme when the transmittedtransmission power is larger than −15 dBm. Based on thhe above observations, we obtain that weather conditions can cause effects on FSO links under the small transmittedtransmission power scenario. Moreover, the BER of the hybrid direct link by the phase modulation scheme is better than that by the frequency modulation. This is because the phase modulation has significant advantages in the environment, where signal suffers from serious attenuations and distortions. From Figure 2, it can be seen that conditions such as clear air, haze, and light fog have a significant impact on the FSO communication links only, while light rain condition has an impact on both FSO and RF communication links. Assuming that the hybrid system is in weather conditions such as atmosphere, haze, and light fog, when the transmission power is low, the BER performance of the hybrid direct links with phase modulation scheme is better than that of frequency modulation. This is because the signal is severely attenuated and distorted during transmission, and phase modulation has a significant advantage in this case.; Moreover, when the transmittedtransmission power is large, the BER of the hybrid direct link by the coherent modulation scheme is better than that by the non-coherent modulation. This is because coherent modulation technology can predict the carrier phase at receivers, which can further improve the system BER when the signal waveform is better. For light rain atmospheric environments, the BER with the phase modulation scheme is better than that of the frequency modulation, and the hybrid direct link with the CBPSK modulation scheme is the best under any weather conditions.

Figure 3 reveals the relationship between the BER and the transmittedtransmission power by the CBPSK modulation scheme under different conditions, such as RF fading parameters $m_{x,y}$*m*, receiver aperture diameters $D_{x,y}$*D*, and weather conditions. Comparing Figure 3a,b, it can be seen that the larger the fading parameter $m_{x,y}$*m*, the lower the BER. The larger the receiver aperture $D_{x,y}$*D*, the more significant improvement of the BER, and the more obvious aperture averaging effect. Besides, from Figure 3, it can be easily observed that the BER is prone to be affected by different weather conditions, where the impact of light fog is the greatest, while the impact of clean air is the smallest. The BERs rapidly decrease when transmittedtransmission powers are larger enough, for example the corresponding transmittedtransmission power at the turning point is also higher for weather with greater light attenuation. This is because when the transmittedtransmission power is low, the main factor affecting the BER is the attenuation of light intensity. Therefore, a limited increase of the transmittedtransmission power will result in a stable decrease in the BER curve; when the transmittedtransmission power becomes larger enough, the main factor affecting the BER changes to the turbulence. Therefore, large transmittedtransmission power will lead to a sharp decrease in the BER. These results indicate that although the BER of the hybrid direct link is significantly affected by both the FSO and RF subchannels, the improvement in any subchannel can significantly improve the BER performance of the hybrid direct link.

In practice, light rain weather condition can have an impact on both FSO and RF links; it would be mandatory to evaluate the BER performance of UAV-assisted hybrid systems under light rain. Figure 4 shows the relationship between the BER and the transmittedtransmission power with CBPSK modulation under light rain for different RF fading parameters $m_{x,y}$*m*, relay-assisted structures, and receiver aperture diameters. From Figure 4a, it can be seen that under the same system conditions, compared to the hybrid direct link, UAV-assisted hybrid systems can significantly improve the BER performance, and the aperture averaging effect can enhance the performance. In Figure 4b, it can be seen that the RF fading parameter $m_{x,y}$*m* plays an important role on the BER performance of the UAV-assisted hybrid system. Specifically, the larger the value of $m_{x,y}$*m*, the smaller the BER. In addition, Figure 4a,b show that different relay-assisted structures have various effects on the UAV-assisted hybrid system. More specific, the structure of (5, 3) has the best BER performance, while (1, 3) is the worst. This is because the increase in the number of transmission path not only enlarges the coverage but also increases the diversity gain, which further reduces the system BER. The increase in the number of transmission hops not only enlarges the distance but also leads to the accumulation of bit error rates, which further increases the system BER.

Figure 5 shows the relationship between the outage probability and transmittedtransmission power for different RF fading parameters $m_{x,y}$*m*, receiver aperture diameter $D_{x,y}$*D*, and weather conditions when the decision threshold is 1 dB. From Figure 5a,b, it can be seen that the fading parameter $m_{x,y}$*m* of the RF sublink dominates the outage probability of the hybrid direct link. This is because (1) the larger the value of $m_{x,y}$*m*, the smaller the outage probability. (2) The larger the receiver aperture $D_{x,y}$*D*, the more improvement of the outage performance. Moreover, it can be easily observed from Figure 5 that the outage probability of hybrid direct links is easily affected by different weather conditions, which can also be corroborated from Figure 3. Similar to Figure 3, outage curves in Figure 5 decrease heavily when transmission powers are larger than a threshold, which are different for different weather conditions.

Figure 6 depicts the relationship between outage probability and the transmittedtransmission power of UAV-assisted hybrid systems and hybrid FSO/RF direct links for different RF fading parameters $m_{x,y}$*m*, weather conditions, relay-assisted structures, and aperture diameters $D_{x,y}$*D* when $\gamma_{th}=1$ dB$r_{th}=1$ dB and a light rain condition is applied. Similar to Figure 4, compared to the hybrid direct link, we have the following observations. (1) The UAV-assisted hybrid system can significantly improve system outage performance; (2) the aperture averaging effect can further improve outage performance; (3) the RF fading parameter $m_{x,y}$*m* of the UAV-assisted hybrid system is also an important factor for the outage performance; (4) more transmission paths will reduce the outage probability, while more hops in each path will increase the outage probability.

**Figure 7:**
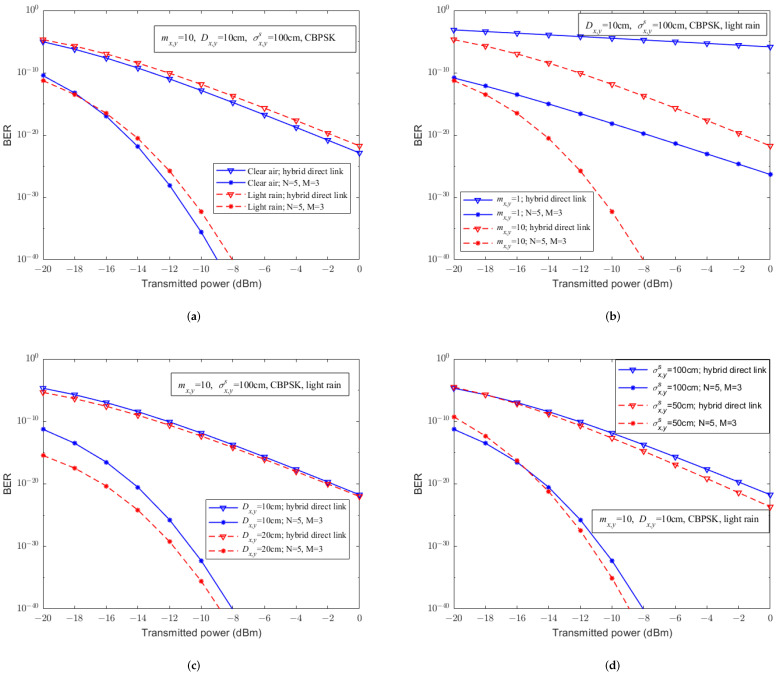
The relationship between BER performance and transmitted power of the hybrid FSO/RF direct links and UAV-assisted hybrid systems with PE under different parameters. (**a**) Different weather. (**b**) Different $m_{x,y}$. (**c**) Different $D_{x,y}$. (**d**) Different $\sigma_{x,y}^{s}$.

Based on the experimental results in Figure 2, Figure 3, Figure 4, Figure 5 and Figure 6 and considering pointing errors, further analysis is conducted on the impact of the proposed system on mitigating the atmospheric environment. Figure 7 shows the relationship between the bit error rate and the transmitted power of UAV-assisted hybrid systems with (5, 3) relay structure and hybrid FSO/RF direct links for different parameters, such as weather, RF fading parameters $m_{x,y}$, receiver apertures $D_{x,y}$, and the standard deviation of the pointing errors on the receiver plane $\sigma_{x,y}^{s}$. According to Figure 7, it can be seen that under the consideration of pointing errors, compared to the hybrid FSO/RF direct link, the UAV-assisted hybrid system significantly improves the BER performance under any conditions. As $m_{x,y}$ increases, $D_{x,y}$ increases, or $\sigma_{x,y}^{s}$ decreases, it can further improve the error rate performance of UAV-assisted hybrid systems and hybrid FSO/RF direct links.

Furthermore, from Figure 7a, we can observe that when the transmitted power is low, the bit error rate performance of UAV-assisted hybrid systems and hybrid FSO/RF direct links under light rain conditions is better than that under clear air, while when the transmitted power is high, this situation is exactly the opposite. Similarly, we also found in Figure 7d that when the transmitted power is low, the bit error rate performance of UAV-assisted hybrid systems and hybrid FSO/RF direct links with $\sigma_{x,y}^{s}=100$ cm is better than that under $\sigma_{x,y}^{s}=50$ cm, and when the transmitted power is high, this situation is also opposite. This is because when the transmitted power is low and the standard deviation of the PE is large or the atmospheric attenuation is large, the RF sublink replaces the FSO sublink as the main method of information transmission, and the error rate performance is actually better. As the transmitted power increases, the SNR of the FSO sublink is higher and the bit error rate performance is rapidly improved.

**Figure 8:**
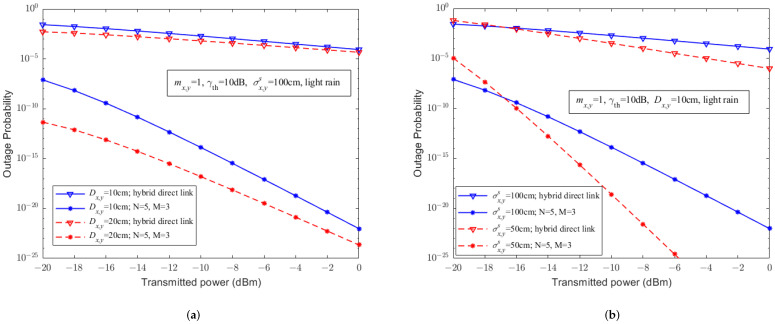
The relationship between outage probability and transmitted power of UAV-assisted hybrid systems and hybrid FSO/RF direct links with PE. (**a**) Different $D_{x,y}$. (**b**) Different $\sigma_{x,y}^{s}$.

When $m_{x,y}=1$, $\gamma_{th}=10$ dB, and light rain, Figure 8 describes the relationship between the outage probability and the transmitted power of UAV-assisted hybrid systems with a (5, 3) relay structure and hybrid FSO/RF direct links for different parameters, such as receiver apertures $D_{x,y}$ and the standard deviation of the pointing error on the receiver plane $\sigma_{x,y}^{s}$. Similar to the conclusion in Figure 7, it can also be seen that under the consideration of pointing errors, compared to the hybrid FSO/RF direct link, the UAV-assisted hybrid system significantly improves the outage performance. As $D_{x,y}$ increases, or $\sigma_{x,y}^{s}$ decreases, it can further improve the outage performance of UAV-assisted hybrid systems and hybrid FSO/RF direct links.

## 5. Conclusions

In this paper, with the consideration of practical factors such as atmospheric loss, atmospheric turbulence, weather conditions, RF channel fading, pointing errors, and relay-assisted structure, we derive mathematical expressions of the BER and outage probability of the hybrid FSO/RF direct link and the UAV-assisted hybrid system, respectively. We, firstly, analyze the impact of different modulation schemes on the BER of both the hybrid direct link and the UAV-assisted hybrid system and conclude that the BER performance can achieve an optimum when adopting the CBPSK modulation scheme under any atmospheric environment and system conditions. Through simulation analyses, we have that the light fog condition has the most severe impact on system performance, while the clear air condition has the least impact on the system. Although the impact of light rain on both FSO and RF sublinks exist, the system performance is still better than that in haze and light fog conditions. More receiver aperture and RF fading parameter $m_{x,y}$*m* can further improve the performance of the hybrid direct link and the UAV-assisted hybrid system under any weather conditions. Compared with the hybrid direct link, the UAV-assisted hybrid system can significantly improve system communication performance. For the UAV-assisted hybrid system, increasing the number of transmission paths will improve communication performance, while fewer hops in each path will contribute to the performance. This work also demonstrates that combining multiple technologies can effectively improve the communication performance of hybrid direct links and UAV-assisted hybrid systems in any weather environment.

## Figures and Tables

**Table 1:** Atmospheric Channel Parameters [30,33,34,35].

Weather Condition	$C_{n}^{2}$ [$m^{-2/3}$]	$\omega_{x,y}^{\mathrm{FSO}}$ [dB/Km]	$\omega_{x,y}^{\mathrm{Rain}}$ [dB/Km]	$\omega_{x,y}^{\mathrm{Oxg}}$ [dB/Km]
Clear air	$5\times 10^{-14}$	0.43	0	15.1
Haze	$1.7\times 10^{-14}$	4.2	0	15.1
Light fog	$3\times 10^{-15}$	7.7	0	15.1
light rain (2.5 mm/h)	$6\times 10^{-15}$	1.98	1.50	15.1

**Table 2:** Parameters *p* and *q* for the various binary modulation scheme [16,39].

Binary Modulation Scheme	*p*	*q*
Coherent binary phase shift keying (CBPSK)	0.5	1
Differential binary phase shift keying (DBPSK)	1	1
Coherent binary frequency shift keying(CBFSK)	0.5	0.5
Non-coherent binary frequency shift keying (NBFSK)(NBPSK)	1	0.5

**Table 3:** The UAV-assisted hybrid FSO/RF system parameters [30,33,34,35].

FSO Subsystem		RF Subsystem	
Parameters	Value	Parameters	Value
Wavelength, $\lambda_{x,y}^{FSO}$	1550 nm	Wavelength of 60 GHz RF, $\lambda_{x,y}^{RF}$	5 mm
Divergence angle, $\theta_{x,y}$	1 mrad	Nakagami fading parameter, $m_{x,y}$	2
Receiver aperture diameter, $D_{x,y}$	10 cm or 20 cm	Transmit antenna gain, $G_{x,y}^{t}$	44 dBi
Responsivity, $R_{x,y}^{FSO}$	0.5 A/W	Receive antenna gain, $G_{x,y}^{r}$	44 dBi
Noise Variance, ${\left(\sigma_{x,y}^{FSO}\right)}^{2}$	$10^{-14}\;A^{2}/\mathrm{Hz}$	Noise Variance, ${\left(\sigma_{x,y}^{RF}\right)}^{2}$	−85 dBm
Transmission distance, $L_{x,y}$	1 km	Oxygen attenuation, $\omega_{x,y}^{oxg}$	15.1 dB/km

## Data Availability

Not applicable.
